# Adherence to risk evaluation and mitigation strategies (REMS) requirements for monthly testing of liver function

**DOI:** 10.7573/dic.212272

**Published:** 2015-02-10

**Authors:** Christopher M Blanchette, Anthony P Nunes, Nancy D Lin, Kathleen M Mortimer, Joshua Noone, Krishna Tangirala, Stephen Johnston, Benjamin Gutierrez

**Affiliations:** 1 University of North Carolina, Charlotte, NC, USA;; 2 Optum Epidemiology, Waltham, MA, USA;; 3 Otsuka America Pharmaceutical, Inc., Princeton, NJ, USA;; 4 Truven Health Analytics, Washington, DC, USA

**Keywords:** risk evaluation, REMS, liver function test, adherence, compliance, FDA, patient assessment, administrative claims

## Abstract

**Background:**

Risk evaluation and mitigation strategies (REMS), as mandated by the US Food and Drug Administration (FDA) for medications with the potential for harm, are increasingly incorporating rigid protocols for patient evaluation, but little is known about compliance with these programs. Despite the inherent limitations, data on administrative claims may provide an opportunity to investigate adherence to these programs.

**Methods:**

We assessed adherence to liver function test (LFT) requirements included in the REMS program for bosentan through use of administrative claims. Patients observed in the Optum Research Database who were initiators of bosentan from November 20, 2001 to March 31, 2013 were included. Adherence to LFTs was calculated using pharmacy claims for bosentan dispensation and medical claims for laboratory services, and was assessed at the time of drug initiation and within specified time intervals throughout follow-up.

**Results:**

Of 742 patients, 523 (70.5%) had ≥1 qualifying LFT. Among patients with ≥12 dispensations, claims for LFTs at individual dispensations were 53.2–64.0%. Median proportion of dispensations with ≥1 LFT was 0.8 among patients with ≥6 (interquartile range, 0.7–1.0) or ≥12 (0.7–0.9) dispensations. Adherence was 90–100% for 33.3% of all initiators, whereas 29.3% of initiators were non-adherent (defined as <50% of on-therapy LFTs).

**Conclusions:**

Analyses of administrative claims suggest that the REMS program for bosentan may not have adequately guaranteed adherence to the program’s monthly monitoring of LFTs. Such investigations of existing REMS programs may provide insight on how to accomplish more successful evaluation of REMS.

## Introduction

An important function of the US Food and Drug Administration (FDA) is to manage and minimize the risk for potential adverse events from approved drugs. FDA Amendments Act of 2007 gave the FDA the authority to mandate incorporation of risk evaluation and mitigation strategies (REMS) for drugs with the potential for harm [1]. All REMS programs must have a “minimal strategy” timetable for assessment at defined intervals, and can also include one or more of the following: medication guide; communication plan; elements to ensure safe use (ETASU); an implementation plan for ETASU [2,3]. The various elements of REMS programs are designed to ensure safe use of a product and can include: restricted access; healthcare provider or pharmacy certifications; regular monitoring of patients [2,3].

The REMS process is evolving, and the lack of standardization often complicates implementation of individual programs [3]. Safest possible use of treatments can be achieved through collaboration of all parties: patient, provider, manufacturer, and the FDA. Recently, the FDA has required REMS programs to include rigid protocols for patient evaluation as a condition for continued dispensation of the covered medication. Protocols may include required laboratory tests and, if pre-specified thresholds are met, termination of the medication is required. These protocols can be valuable in reducing risk to patients but only if they are targeted successfully toward, and adhered to by, the particular patient population at risk for specific adverse events, along with the treating physician. To ensure that the precautions put in place by REMS are being implemented appropriately, adherence to their requirements should be monitored and, if necessary, actions should be taken to improve deficiencies. Little is known about adherence to REMS programs by patients and providers [4]. The present study was designed to evaluate the level of adherence to specific requirements within a rigid REMS program.

Bosentan is an endothelin receptor antagonist indicated for treatment of pulmonary arterial hypertension (PAH) [5]. Bosentan has one of the most restrictive REMS programs of any medication on the market, including multiple requirements regarding potential risks for hepatotoxicity and birth defects [6]. Bosentan is available only through the Tracleer (bosentan) Access Program (TAP), which requires provider attestation of liver function testing before initiation of bosentan use and patient attestation of monthly assessments during therapy. One element of REMS and the TAP requires providers to have reviewed pretreatment liver function tests (LFTs) and ordered monthly LFTs for anticipated duration of therapy (≤1 year) before first prescribing bosentan to a patient. Despite these requirements for bosentan, a “Dear Doctor” letter was issued in 2006 to report labelling changes based on rare cases of hepatotoxicity as well as to remind providers about the importance and frequency of required LFTs [7]. A more stringent modification of REMS was issued in 2012, which added elements to improve adherence with LFT assessments [6].

The bosentan REMS program was evaluated in the present study because its rigid protocols for patient assessment and medication access may serve as a model for future program development. In an effort to assess adherence to LFT within this REMS program and changes in adherence after communications and actions regarding regulation of drug safety, we used data based on administrative claims (a common data source for pharmacoepidemiologic studies and evaluation of drug safety).

## Methods

### Study design

This observational study used data regarding eligibility, pharmacy, and medical claims from the Optum Research Database (ORD), representing patients from a large US health plan. The ORD is a proprietary database with geographically diverse enrollment data from 1993 to the present day. Data relating to ≈12.6 million individuals with medical and pharmacy benefit coverage are available for 2012.

**Figure 1:**
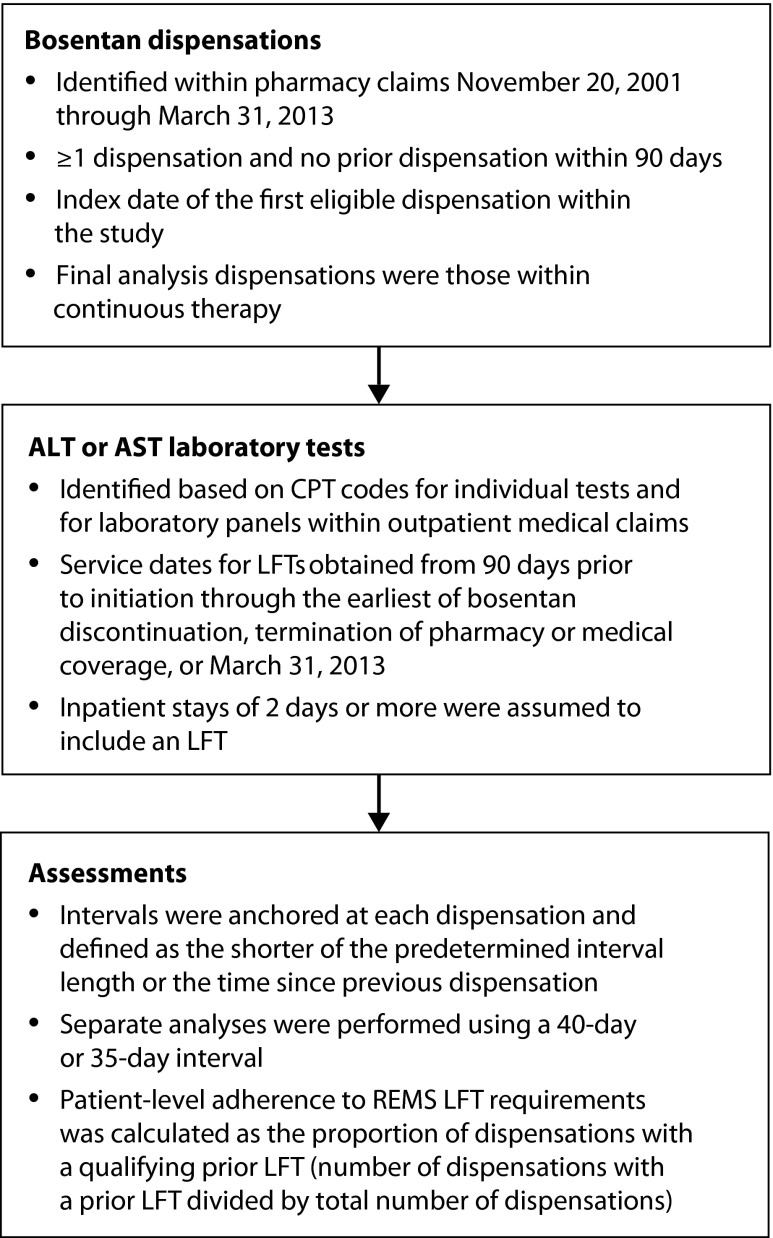
Study methodology. ALT, alanine amino transferase; AST, aspartate aminotransferase; CPT, current procedural terminology; LFT, liver function test; REMS, risk evaluation and mitigation strategies; TAP, Tracleer (bosentan) Access Program.

Study population comprised a cohort of patients who received at least one dispensation of bosentan between November 20, 2001 and March 31, 2013 (Figure 1). Patients must have had ≥90 days of medical coverage and pharmacy benefits before cohort entry. Patients also had to be aged <65 years and have no additional public or private insurance. Bosentan dispensations were identified within pharmacy claims through Hierarchical Ingredient Codes Lists. Initiators of bosentan were defined as those with ≥1 pharmacy claim for dispensation of bosentan and no prior dispensation within 90 days. The index date was the date of the first eligible dispensation within the study period. Patients were followed up until the earliest of the following: discontinuation of bosentan therapy, disenrollment, or the end of the study period (March 31, 2013). Discontinuation of bosentan was assumed if the gap between refills exceeded the days supplied plus a 10-day grace period. Therefore, the period of observation for each individual was restricted to the first interval of continuous bosentan therapy.

LFT characterization included laboratory claims for aspartate aminotransferase (AST) and alanine aminotransferase (ALT), as well as for panels that included AST or ALT. Assessments attempted to replicate the monitoring requirements set forth in the bosentan REMS program in how the measure was defined, but the timing was varied conservatively [6]. Laboratory tests for levels of AST and ALT were identified on the basis of Current Procedural Terminology codes for individual tests and for laboratory panels present in outpatient medical claims.

Laboratory panels containing ALT and/or AST are commonly ordered during inpatient stays. However, data on inpatient claims do not reliably contain information on specific laboratory tests due to the bundling of service charges. Therefore, inpatient stays of ≥2 days were assumed to include a LFT.

To assess adherence, patient follow-up was divided into mutually exclusive intervals anchored at the time of each bosentan dispensation. Interval lengths were defined as the shorter of either 40 days before dispensation or the time since the previous dispensation. Sensitivity analyses were undertaken using a 35-day interval. LFT adherence associated with the index dispensation of bosentan was assessed over a 90-day interval. Adherence to LFT requirements within REMS was calculated using pharmacy claims for bosentan dispensation and medical claims for laboratory services. Binary indicators of ≥1 LFT were generated for the index dispensation and each subsequent refill. Within patients, adherence to guidelines for LFT monitoring was calculated as the number of bosentan dispensations with an associated prior LFT divided by the total number of bosentan dispensations.

### Analyses

Distributions of LFT adherence were analyzed overall and observed by time periods of interest. Analyses were conducted using SAS^*^ 9.2. Distributions were compared, but no formal tests were conducted to evaluate determinants of adherence by statistical means. Reported measures included proportions, means, and standard deviations (SDs), as well as medians and interquartile ranges (IQRs). Characteristics of the study population that were summarized included: demographics; type of provider of index dispensation; medical utilization; medical diagnoses during baseline and follow-up.

Adherence classifications were defined as “high” (90–100%), “moderate” (75–89%), “low” (50–74%), and “non-adherent” (<50%). Adherence per year was evaluated by year of index dispensation (adherence per patient was categorized into the year of the patient’s date of initiation). Regulatory events of interest during the study period included: a Dear Doctor letter issued in March 2006; expansion of indications in August 2009 to include milder forms of PAH; REMS modification in October 2012 to add elements to improve compliance with LFT assessments.

## Results

### Characteristics of the study and Patients

The study population consisted of 742 patients in whom bosentan therapy had been initiated (Table 1). Consistent with the indications for bosentan, most patients had a diagnosis of chronic pulmonary heart disease or PAH. Most prescribing providers were cardiologists or pulmonologists. Patients were admitted for a mean of 3 inpatient days, which is consistent with the severity of the primary indication for bosentan. Median number of bosentan dispensations was 4 (IQR, 1.0–9.0) and the distribution time between consecutive dispensations centered on 30 days. Median time between bosentan dispensation and a prior LFT was longer for the index dispensation compared with the median time between dispensation and a prior LFT for the first 12 refills (Table 2). However, assessment of the index dispensation was defined as a 90-day interval compared with the 40-day interval for refill dispensations. Mean number of days between dispensation and the most proximal prior LFT was 10.0–13.6.

### Adherence to LFTs

Of the 742 patients, 523 (70.5%) had at ≥1 qualifying LFT before the index dispensation (Table 3). In the 40-day assessment, among patients with ≥12 dispensations, the percentage of patients with a claim for a LFT was lowest at dispensation number 8 (53.2%) and highest at dispensation number 3 (64.0%). Adherence (defined for each person as the median proportion of dispensations with ≥1 LFT) was 0.8 among patients with ≥6 (IQR 0.7–1.0) or ≥12 (0.7–0.9) dispensations. Among all initiators, 33.3% had high adherence (90–100% of dispensations had a corresponding LFT), whereas 29.3% of all initiators were considered to be non-adherent (<50% of bosentan dispensations had a corresponding LFT). The level of adherence was similar whether the assessment used 40- or 35-day intervals.

**Figure 2:**
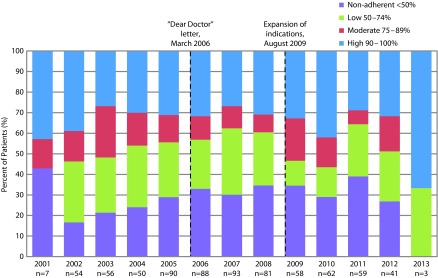
Temporal changes in adherence by calendar year^*^ of the index date and by adherence periods of LFTs as defined by FDA inquiries and action^†^ within the Optum Research Database from November 20, 2001 to March 31, 2013. ^*^Based on 40-day intervals; adherence per patient was categorized into the year of the patient’s date of initiation. ^†^Regulatory events of interest during the study period include a Dear Doctor letter issued in March 2006 and expansion of indications to include milder forms of PAH in August 2009. FDA, Food and Drug Administration; LFT, liver function test; REMS, risk evaluation mitigation strategies.

Aside from years 2001 and 2013 (for which patient numbers were low), annual classification of adherence was: 26.8–41.9% for patients classified as highly adherent; 6.8–25.0% for those classified as moderately adherent; 14.5–32.3% for those classified as low adherent; 16.7–39.0% for those classified as non-adherent (Figure 2). Among patients considered to be highly adherent, adherence was highest among those who initiated in the years immediately after the initial approval for bosentan, with another peak for those initiating in 2010, for which 41.9% patients were considered to be highly adherent. Changes in LFT adherence were not observed after identified events of interest, including a Dear Doctor letter reminding providers about the importance and frequency of required LFTs (March 2006) and an expansion of indications to include milder forms of PAH (August 2009).

## Discussion

In general, REMS initiatives are imperative to ensuring patient safety. Investigation of individual REMS programs can lead to better future programs with the potential to increase patient safety, and permit use of medications that may confer benefit in select populations despite inherent risk. Adherence by physicians and patients to specified requirements is critical to the effectiveness of a REMS program. Hence, we investigated adherence to a restrictive REMS program implemented for bosentan. The REMS program for bosentan is a model program in terms of complexity and requirements. However, when based on evaluation of data of administrative claims, these findings suggest that providers and patients may not fully adhere to these requirements.

An early study following the mandate to authorize the FDA to implement REMS revealed significant concerns among stakeholders from various perspectives – healthcare providers, drug sponsors, patient advocates, payers, and pharmacists – that REMS would have significant impacts on the healthcare system [2]. Current studies highlight the steps being proposed and taken to improve REMS programs, particularly to standardize programs and evaluate effectiveness [3,4]. The FDA recently introduced a REMS Integration Initiative to evaluate and improve implementation of REMS programs [8].

Several studies have also begun to analyze various aspects of the FDA REMS program. Secular trends in REMS approval, assessment of approved REMS characteristics, and time lags between drug approval and REMS approval were investigated through data collected from FDA Approved Drug Products with Therapeutic Equivalence Evaluations [9]. That particular study reported FDA-approved REMS for 1 in 3 biologics and 1 in 13 chemical entities available in the US market, and that the number of pharmaceuticals with serious risk requiring REMS increased significantly over time. However, recent policy changes by the FDA with regard to some medication guides not requiring REMS indicate that a full REMS will be reserved for a small number of high-risk medications [10]. The International Society for Pharmacoeconomics and Outcomes Research Risk Benefit Management Working Group analyzed and compared the FDA’s REMS program and the European Medicines Agency’s Risk Management Plan [11]. They found that both provide positive guidance for identifying, monitoring, and minimizing risks to patient safety, but neither provides specific guidance on how risk should be balanced with benefit, either qualitatively or quantitatively [11]. Risk communication is an integral part of REMS programs, but the educational effectiveness of the approach has not been studied extensively. A recent investigation analyzed the approach of adding an Internet-based continuing-medical-education activity, and found improvement in the understanding of specific drug toxicities among healthcare providers [12].

In the present study, adherence to an example of a stringent program that requires LFT monitoring was investigated utilizing only data on administrative claims. Adherence to REMS requirements for LFT monitoring while on continuous therapy was 70.5%. In the context of the present study, the evidence did not suggest meaningful improvement in adherence following periods defined by FDA inquiries and actions pertaining to LFTs. As sensitivity analyses, data from another database on administrative claims representing a different patient population were assessed. The Truven Health Marketscan Commercial administrative claims database comprises administrative claims from ≈40 million employees and dependents covered annually under various health insurance plans. Among 660 patients with a bosentan dispensation between October 1, 2009 and September 30, 2012, adherence to LFTs within 35 days before dispensation was 50.3% in patients with ≥1 dispensation (data not shown). Adherence increased to 62% if considering inpatient stays as unobserved (but potentially valid) LFT events. For patients who underwent all 12-treatment cycles (*n*=228), 61.4% were adherent; this value increased to 64.9% with incorporation of tests during hospitalization. Adherence levels observed in these sensitivity analyses were slightly lower than those observed using the ORD.

It is clear that an ongoing tracking system can provide more accountability and reconciliation of adherence with REMS programs due to the inability of readily available data sources to completely capture non-adherence by patients and physicians. Studies to evaluate these systems are becoming more prevalent. For example, a study covering 2005–2011 for evaluation of asthma medications found that the implemented REMS program resulted in a reduction of use of fluticasone propionate/salmeterol and encouraged appropriate use of long-acting B_2_-adrenergic agonists, a major goal of the REMS program [13]. Even with manufacturers and the FDA working together to develop REMS programs to ensure patient safety, managing the risk effectively without full engagement of the patient and provider (as well as a method to track such an engagement) is difficult.

Investigation of REMS programs (and especially adherence to their measures) is necessary. Databases of administrative claims (such as those used in our study) are a convenient and cost-effective method of investigation. However, interpretation of findings based solely on electronic claims should consider the inherent limitations in the processing and accumulation of these data. Claims for LFTs were observed, but claims data do not enable assessment of whether LFTs were reviewed by the provider, or whether the provider responded appropriately to elevated laboratory results, which is required by the REMS and which would provide more complete analyses of adherence. In addition, claims-based data include medication dispensations, but there is no assurance that the medication was taken as prescribed. Furthermore, the ORD includes a large and diverse patient population, but patients within this analytic dataset may not fully represent the patterns of adherence observed in the general population.

A LFT claim is strongly indicative that an LFT occurred, but additional variability may be present due to unobserved testing. In the present study, data for patients aged >65 years were excluded, and results were limited to the subset that was commercially insured without dual coverage from other insurers (government, commercial, private). This restriction was intended to limit the possibility of observing a claim for a dispensation of bosentan within the ORD among patients for whom laboratory testing is covered by an alternative insurer (and therefore not observed as a claim within the ORD). Consistent with this hypothesis, sensitivity analyses involving patients with co-insurance yielded notably lower estimates of adherence. The restriction of dual insurance limited the possibility of unobserved fulfillment of testing requirements, but the possibility remains that patients could have received tests in another manner (e.g., *via* manufacturer-sponsored programs and free clinics), which is a limitation of use of claims data.

Investigating existing REMS programs with rigid protocols may provide insight on how to accomplish more successful implementation of REMS with similar restrictions. Taking into account the limitations of using claims data, the results of this study indicate less-than-optimal adherence. Joint efforts between the FDA and pharmaceutical companies (including efforts to increase adherence from patients and providers) may make improvement of REMS more successful.

## Figures and Tables

**Table 1:** Demographic characteristics of bosentan initiators within the Optum Research Database from November 20, 2001 to March 31, 2013.

**n (%)**	**All initiators (N=742)**	**Initiators with ≥12 dispensations (n=139)**
Age at initiation, years		
<18	58 (7.8)	10 (7.2)
18–29	44 (5.9)	6 (4.3)
30–39	76 (10.2)	18 (13.0)
40–49	180 (24.3)	29 (20.9)
50–59	248 (33.4)	48 (34.5)
60–65	136 (18.3)	28 (20.1)

Sex		
Male	212 (28.6)	41 (29.5)
Female	530 (71.4)	98 (70.5)

Geographic area		
Northeast	53 (7.1)	8 (5.8)
Midwest	207 (27.9)	39 (28.1)
South	382 (51.5)	69 (49.6)
West	100 (13.5)	23 (16.6)

Provider of the index prescription		
Cardiologist	155 (20.9)	32 (23.0)
Pulmonologist	365 (49.2)	70 (50.4)
Rheumatologist	38 (5.1)	6 (4.3)
Generalist	45 (6.1)	6 (4.3)
Other	49 (6.6)	7 (5.0)
Unknown	90 (12.1)	18 (13.0)

Diagnosis while on therapy		
Chronicpulmonary heart disease (416.x)	547 (73.7)	98 (70.5)
Pulmonaryartery hypertension (416.0)	486 (65.5)	91 (65.5)
Acute liver failure	3 (0.4)	0 (0.0)

Measures of healthcare utilization during follow-up		
Number of outpatient visits, mean (SD)	4.5 (5.2)	3.7 (4.8)
Number of inpatient days, mean (SD)	3.0 (8.8)	1.8 (5.0)
Number of laboratory tests, mean (SD)	3.0 (3.6)	2.7 (3.0)

Number of continuous on-therapy dispensations		
Mean (SD)	7.6 (10.8)	NA
Median (SD)	4.0 (1.0, 9.0)	NA
Days between dispensation		
Mean (SD)	29.7 (5.2)	NA
Median (SD)	30.0 (27.0, 33.0)	NA

NA, not available; SD, standard deviation.

**Table 2:** Characterization of bosentan dispensations and relative timing of liver function tests within the Optum Research Database from November 20, 2001 to March 31, 2013.

	**Patients with dispensation**	**Patients with a LFT within the previous 40 days**	**Days between dispensation and most proximal prior LFT**	
	**(n)**	**(n)**	**Mean (SD)**	**Median (p25, p75)**
Index dispensation^*^	742	523	26.1 (22.9)	18.0 (8.0, 40.0)

1st refill	538	285	10.0 (8.3)	8.0 (3.0, 16.0)

2nd refill	469	265	12.4 (8.9)	11.0 (5.0, 19.0)

3rd refill	394	237	12.8 (9.2)	12.0 (4.0, 20.0)

4th refill	334	193	12.6 (9.2)	12.0 (4.0, 20.0)

5th refill	290	182	13.6 (8.9)	13.0 (6.0, 20.0)

6th refill	251	150	13.2 (9.3)	12.0 (5.0, 20.0)

7th refill	226	139	13.4 (9.2)	13.0 (6.0, 21.0)

8th refill	209	117	12.3 (9.3)	11.0 (5.0, 20.0)

9th refill	184	106	13.0 (9.0)	13.0 (6.0, 20.0)

10th refill	147	87	12.7 (8.8)	12.0 (6.0, 20.0)

11th refill	139	85	11.8 (9.3)	9.0 (4.0, 18.0)

12th refill	129	73	12.6 (9.4)	10.0 (4.0, 21.0)

* Within a 90-day interval for the index dispensation.

LFT, liver function test; SD, standard deviation.

**Table 3:** Assessment of adherence^*^ to monitoring of liver function within the Optum Research Database from November 20, 2001 to March 31, 2013.

	**All initiators (N=742)**	**Initiators with ≥12 dispensations (n=139)**
**≥1 LFT within dispensation-specific assessment intervals, n (%)**		

Index dispensation	523 (70.5)	99 (71.2)
1st refill	285 (53.0)	77 (55.4)
2nd refill	265 (56.5)	84 (60.4)
3rd refill	237 (60.2)	89 (64.0)
4th refill	193 (57.8)	83 (59.7)
5th refill	182 (62.8)	81 (58.3)
6th refill	150 (59.8)	79 (56.8)
7th refill	139 (61.5)	81 (58.3)
8th refill	117 (56.0)	74 (53.2)
9th refill	106 (57.6)	79 (56.8)
10th refill	87 (59.2)	80 (57.6)
11th refill	85 (61.2)	85 (61.2)
12th refill	73 (56.6)	73 (56.6)

**Proportion of dispensations with ≥1 prior LFT while continuously on therapy**		

All dispensations		
Mean (SD)	0.6 (0.4)	0.6 (0.3)
Median (p25, p75)	0.7 (0.3, 1.0)	0.6 (0.3, 0.8)
First 6 dispensations^†^		
Mean (SD)	0.8 (0.2)	0.7 (0.2)
Median (p25, p75)	0.8 (0.7, 1.0)	0.7 (0.7, 0.8)
First 12 dispensations^‡^		
Mean (SD)	0.8 (0.2)	0.8 (0.2)
Median (p25, p75)	0.8 (0.7, 0.9)	0.8 (0.7, 0.9)

**Categories of LFT adherence**		

High: 90–100% of on-therapy LFTs	247 (33.3)	15 (10.8)
Moderate: 75–89% of on-therapy LFTs	105 (14.2)	35 (24.2)
Low: 50–74% of on-therapy LFTs	173 (23.3)	41 (29.5)
Non-adherent: <50% of on-therapy LFTs	217 (29.3)	48 (34.5)

* Based on 40-day intervals except for the index dispensation, which was a 90-day interval.

† Restricted to patients with ≥6 dispensations of bosentan.

‡ Restricted to patients with ≥12 dispensations of bosentan.

LFT, liver function test; SD, standard deviation.

## Footnotes

* SAS Institute Inc., Cary, NC, USA.

## Abbreviations

ALT: alanine aminotransferase;
AST: aspartate aminotransferase;
ETASU: elements to ensure safe use;
FDA: Food and Drug Administration;
IQR: interquartile range;
LFT: liver function test;
ORD: Optum Research Database;
PAH: pulmonary arterial hypertension;
REMS: risk evaluation and mitigation strategies;
SD: standard deviation;
TAP: Tracleer (bosentan) Access Program
